# The Treatment of Spina Bifida

**Published:** 1887-06

**Authors:** 


*The Treatment of Spina Bifida.* By JAMES MORTON, M.D. Pp. 228. With plates. 2nd Edition. London: J. & A. CHURCHILL. 1887.

Professor Morton's is essentially *the* treatment of Spina Bifida, in the sense of being better than all others; and we are glad to have a full and authoritative account of it provided by the originator. The work is enriched by the addition of Professor Humphry's drawings, and by a special account of the pathology of spina bifida from the pen of Professor Cleland. The author modestly refers to the report of the Clinical Society on his mode of treatment with some gratitude, and at the same time with a certain amount of independent criticism. Special committees have often been appointed by London Societies to enquire into special subjects: in this, as in other instances, they have been very harmless and very useless. The committee is to the original worker what the accountant is to the financier: a prosaic and mechanical critic of independent flights of genius. The profession cares nothing whatever for the committee of the Clinical Society: one independent and practical member of the profession is just as trustworthy as a whole subsidised and theoretical committee; and fortunately his evidence is of more weight with work-a-day men. In the whole history of the progress of human knowledge we have never heard of an original investigation, and rarely of a sound conclusion, being achieved by a committee: it is a combination of impracticables, and among practical men may safely be ignored. We sympathise with Dr. Morton in having to consider the report of this committee of the Clinical Society, and we heartily endorse his dissension from their conclusions.

